# Twiddler’s syndrome resulting in right phrenic nerve stimulation: a case report

**DOI:** 10.1093/ehjcr/ytag175

**Published:** 2026-03-10

**Authors:** Sayyed Jalawan Asjad, Parag Patel, Alexander Somerville, Mark Charlamb

**Affiliations:** Department of Internal Medicine, SUNY Upstate Medical University, 750 E Adams Street, Syracuse, NY 13210, USA; Department of Internal Medicine, SUNY Upstate Medical University, 750 E Adams Street, Syracuse, NY 13210, USA; Department of Cardiology, SUNY Upstate Medical University, 750 E Adams Street, Syracuse, NY 13210, USA; Department of Cardiology, SUNY Upstate Medical University, 750 E Adams Street, Syracuse, NY 13210, USA

**Keywords:** Twiddler’s syndrome, Cardiac implantable electronic device, Phrenic nerve stimulation, Diaphragmatic contractions, Lead displacement, Implantable cardioverter-defibrillator, Case report

## Abstract

**Background:**

Phrenic nerve stimulation is an uncommon complication of cardiac implantable electronic devices (CIEDs), and right-sided stimulation is particularly rare. Twiddler’s syndrome causes lead retraction that may result in extracardiac stimulation.

**Case summary:**

An 87-year-old man with a dual-chamber implantable cardioverter-defibrillator (ICD) presented with 5 days of persistent upper abdominal contractions. Examination showed rhythmic diaphragmatic spasms synchronized with ventricular pacing spikes. Electrocardiography revealed ventricular pacing spikes without myocardial capture. Chest radiograph demonstrated right ventricular (RV) lead displacement into the superior vena cava, consistent with Twiddler’s syndrome. Temporary reprogramming to AAI pacing eliminated symptoms, and definitive RV lead revision restored normal device function. Although the patient did not attend in-person follow-up due to relocation to another state, communication with the patient’s family approximately 1 month after discharge confirmed no recurrence of diaphragmatic spasms.

**Discussion:**

This case demonstrates a rare mechanism of right phrenic nerve stimulation due to cranial migration of an ICD RV lead. Recognition and timely device reprogramming are essential for the resolution of diaphragmatic spasms.

Learning points

- Cardiac device-related complications should be considered in patients presenting with unexplained abdominal or diaphragmatic spasms.
- Temporary device reprogramming can serve both as a diagnostic tool and an effective therapeutic intervention.

## Introduction

Phrenic nerve stimulation is an uncommon but clinically significant complication following implantation of cardiac implantable electronic devices (CIEDs). Most reported cases involve the left phrenic nerve due to the anatomical proximity between the left ventricular wall and the left phrenic nerve, particularly in cardiac resynchronization therapy where left ventricular leads are positioned in the coronary sinus.^1,2^ In contrast, right phrenic nerve stimulation is rare and typically occurs in the setting of right ventricular (RV) lead perforation or migration into the superior vena cava (SVC).^3^

Twiddler’s syndrome, first described in 1968, is a rare cause of lead displacement resulting from manipulation or rotation of the pulse generator within its subcutaneous pocket.^4^ This rotation leads to lead coiling, retraction, or cranial migration, causing pacing failure or unintended stimulation of adjacent structures.^5^ Although Twiddler’s syndrome is well described, its association with right-sided phrenic nerve stimulation due to RV lead migration into the SVC has only been reported in isolated cases.^6^

We report a unique case of persistent right diaphragmatic spasms caused by RV lead displacement into the proximal SVC secondary to Twiddler’s syndrome in an elderly patient with a dual-chamber implantable cardioverter-defibrillator (ICD). This case highlights an exceedingly rare presentation and illustrates the diagnostic and therapeutic importance of device reprogramming.

## Summary figure

**Figure:**
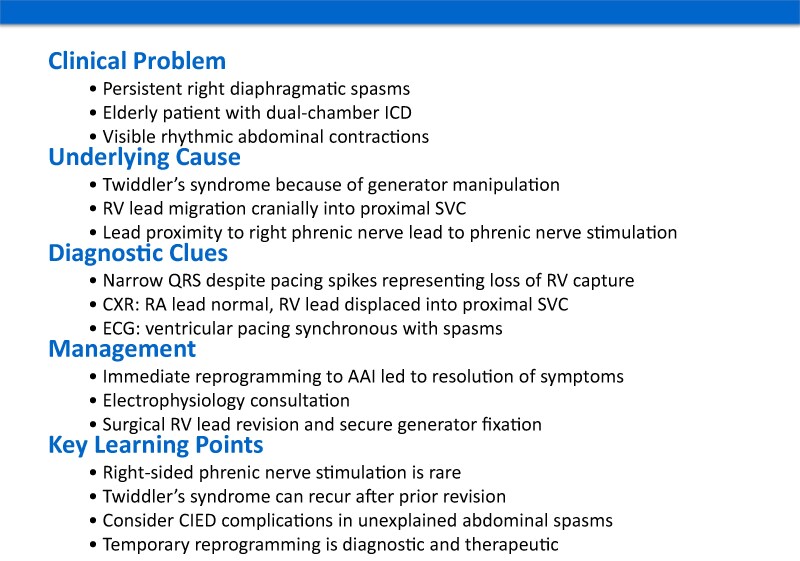


## Case presentation

An 87-year-old man presented to the emergency department with 5 days of persistent upper abdominal spasms. He denied chest pain, nausea, vomiting, reflux symptoms, or dyspnoea. His medical history was notable for type 2 diabetes mellitus, hypertension, hyperlipidaemia, chronic obstructive pulmonary disease, prior ischaemic stroke, and coronary artery disease.

Nine years earlier, he had undergone percutaneous coronary intervention (PCI) to the right coronary artery, with treatment of in-stent restenosis 1 year prior. Nine months before the current presentation, he was admitted with chest pain and subsequently developed a ventricular tachycardia arrest in the emergency department, from which he was successfully resuscitated. Coronary angiography demonstrated a 50%–70% mid left anterior descending artery lesion, which was treated with PCI. Given the ventricular arrhythmia, a dual-chamber ICD was implanted during the same admission for secondary prevention. His underlying rhythm prior to ICD implantation was sinus rhythm. A lead displacement occurred 1 day after implantation, requiring lead revision during that hospitalization.

The most recent transthoracic echocardiogram, performed approximately 5 months prior to the current presentation, demonstrated preserved left ventricular systolic function with an ejection fraction of 60%–65%. No repeat echocardiogram was performed during this admission.

**Figure 1:**
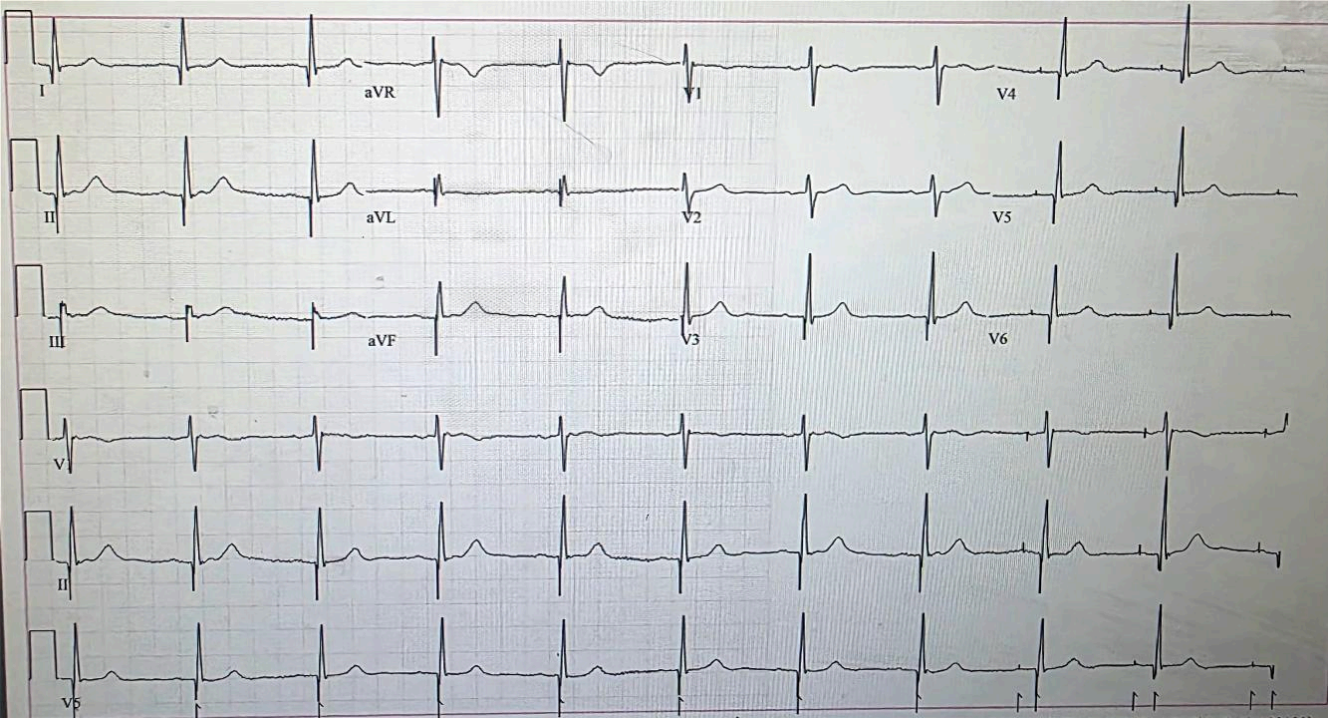
Twelve-lead electrocardiogram at presentation showing paced rhythm associated with diaphragmatic contractions.

On presentation, physical examination revealed visible, rhythmic contractions of the upper abdomen at the thoracoabdominal junction, consistent with diaphragmatic spasms. A 12-lead electrocardiogram showed ventricular pacing spikes preceding each QRS complex, with narrow QRS morphology, indicating pacing output without myocardial capture and preserved intrinsic atrioventricular conduction (*Figure 1*).

**Figure 2:**
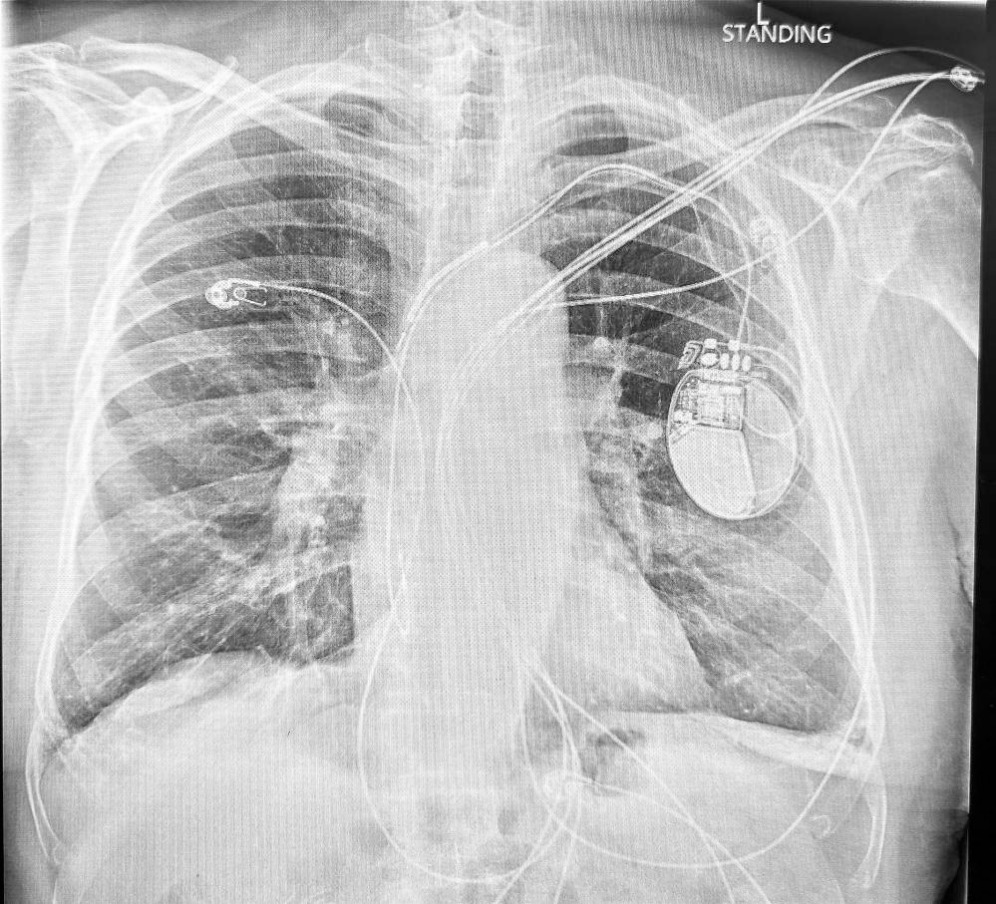
Chest radiograph demonstrating right ventricular lead displacement and coiling into the proximal superior vena cava.

Chest radiography demonstrated appropriate positioning of the right atrial lead, while the RV lead had migrated cranially into the proximal SVC (*Figure 2*). Based on the temporal relationship between pacing activity and symptoms, cardiology evaluation concluded that the abdominal spasms were most consistent with right phrenic nerve stimulation secondary to RV lead displacement.

**Figure 3:**
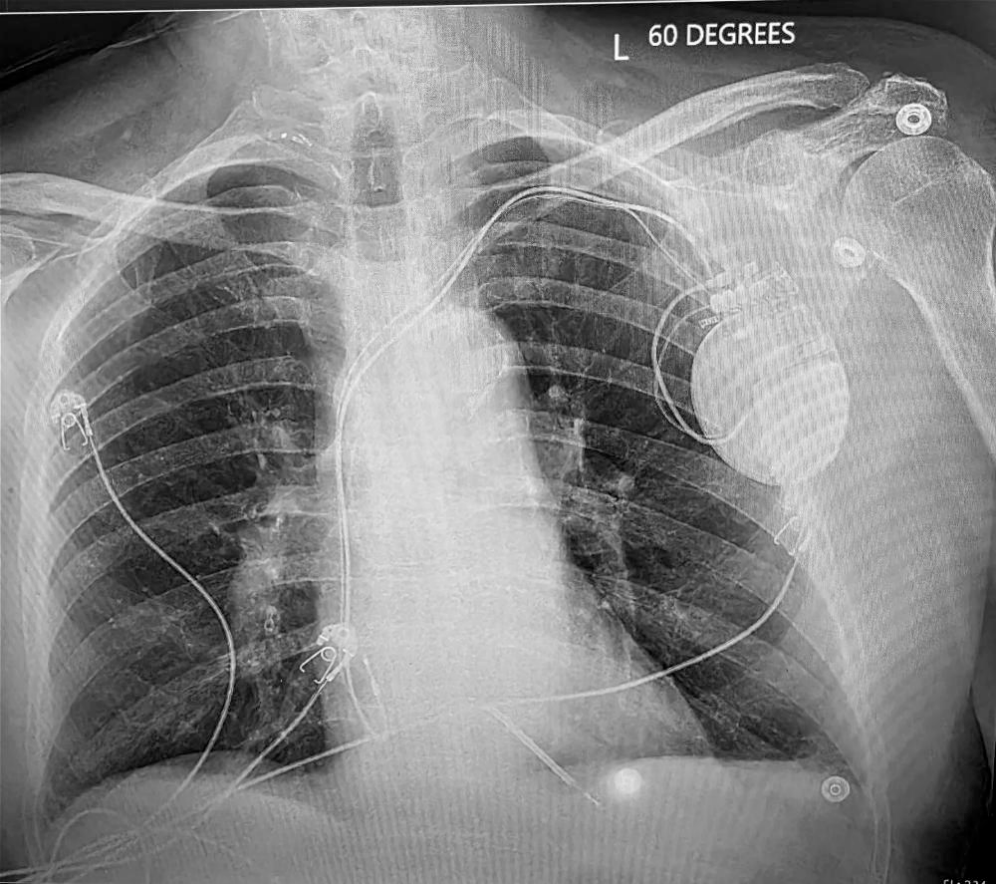
Post-implantable cardioverter-defibrillator replacement chest radiograph showing correctly positioned right atrial and right ventricular leads.

The ICD was temporarily reprogrammed from DDD mode to AAI pacing, resulting in immediate resolution of the diaphragmatic spasms. The patient remained clinically stable on telemetry monitoring. Electrophysiology consultation recommended definitive lead revision, and the patient subsequently underwent successful RV lead repositioning with secure generator fixation (*Figure 3*). During evaluation, the patient acknowledged a habit of fidgeting with the device pocket and was counselled to avoid manipulation of the generator following revision.

**Figure 4:**
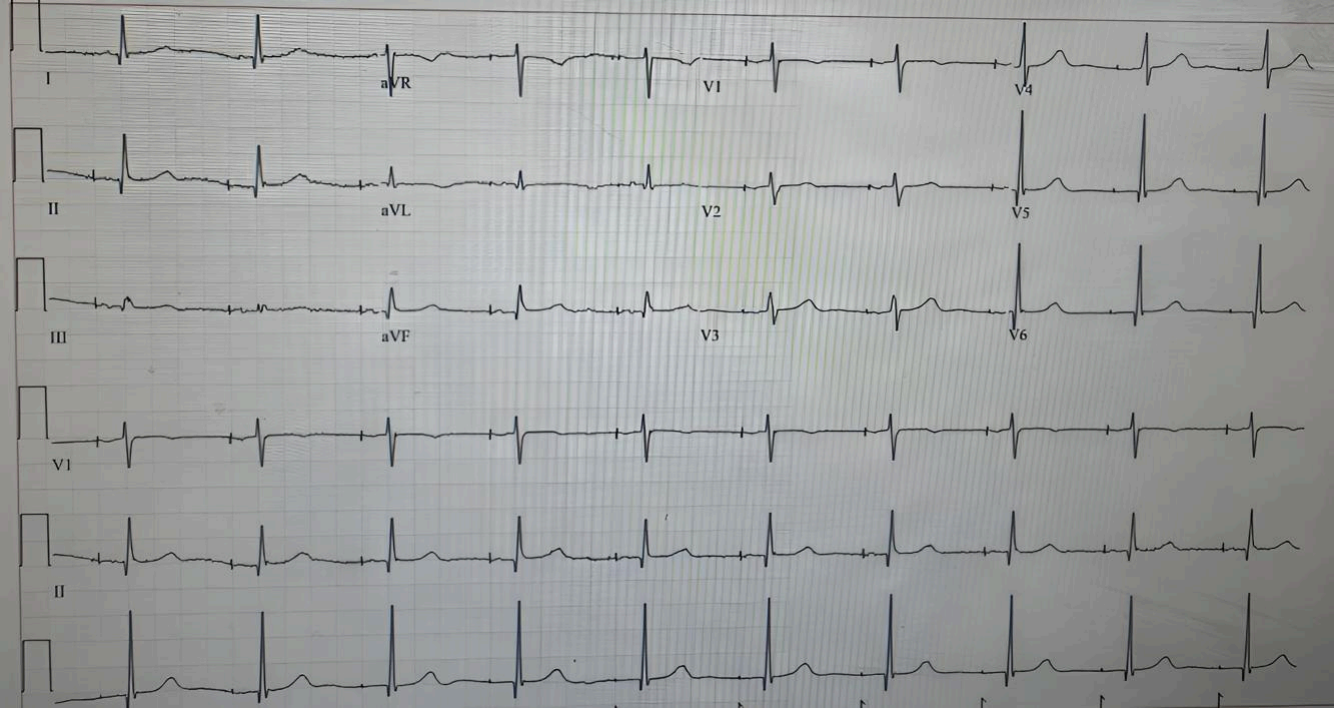
Post-lead replacement electrocardiogram showing resolution of ventricular pacing spikes.

After the procedure, ICD programming was restored to standard settings, and no recurrence of symptoms was observed during the remainder of the hospital stay. Post-lead replacement electrocardiogram showed resolution of ventricular pacing spikes (*Figure 4*). The patient was discharged in stable condition. Although he did not attend in-person follow-up due to relocation to another state, communication with his family approximately one month after discharge confirmed no recurrence of diaphragmatic spasms or related symptoms.

## Case discussion

Twiddler’s syndrome is a recognized mechanical cause of lead displacement, resulting from rotation of the pulse generator within its subcutaneous pocket. This process may lead to lead coiling, retraction, or cranial migration, with subsequent pacing failure or unintended extracardiac stimulation.^5,7^

This case illustrates a rare and clinically important cause of right diaphragmatic stimulation resulting from CIED malfunction. Previous reports have described phrenic nerve stimulation related to displaced leads. Antonelli *et al*.^6^ reported transient abdominal contractions caused by Twiddler’s syndrome with lead retraction to the SVC–right atrial junction. Perez *et al*.^3^ described diaphragmatic stimulation resulting from a displaced ICD lead contacting the right phrenic nerve. Celik *et al*.^8^ reported persistent hiccups secondary to late lead perforation with resultant phrenic nerve activation.

Our case differs from prior reports in several important aspects. First, the patient had a dual-chamber ICD rather than a pacemaker, and the RV lead had migrated higher into the proximal SVC, placing the lead tip in even closer proximity to the anatomical course of the right phrenic nerve. This pronounced cranial displacement likely contributed to the persistent and rhythmic diaphragmatic contractions, which were visibly appreciable on physical examination. Second, the patient had previously undergone lead revision, highlighting that Twiddler’s syndrome may recur even after corrective intervention and underscoring the importance of meticulous generator fixation and comprehensive patient education to reduce recurrence risk. Third, this case demonstrates the clinical utility of temporary device reprogramming, which provided immediate symptom relief and confirmed the pacing-induced aetiology prior to definitive lead revision. Finally, the presence of continuous abdominal contractions synchronized with ventricular pacing emphasizes that device-related causes must be considered in patients presenting with unexplained diaphragmatic or abdominal wall movements.

The mechanism of phrenic nerve stimulation in this patient was both mechanical and electrical. The retracted RV lead, displaced into the SVC, came into close proximity with the right phrenic nerve. Pacing output delivered at this site depolarized the nerve, resulting in rhythmic diaphragmatic contraction with each ventricular pacing impulse. Such presentations may mimic gastrointestinal or neuromuscular disorders and can lead to unnecessary testing or misdiagnosis if CIED-related complications are not included in the differential diagnosis.^3,6^

Diagnosis of Twiddler’s syndrome relies on recognition of key clinical features, including visible muscle contractions, abnormal pacing patterns on electrocardiography, and radiographic evidence of lead coiling or cranial migration. Management involves temporary deactivation or reprogramming of the offending lead, followed by prompt surgical correction. Securing the generator to the pectoral fascia reduces the risk of recurrence, and patient counselling to avoid manipulation of the device pocket is essential.^7^

This case reinforces the importance of considering Twiddler’s syndrome in any patient with a CIED who presents with new-onset diaphragmatic spasms, particularly when symptoms correlate with pacing activity. Although exceedingly rare, right phrenic nerve stimulation due to RV lead displacement into the proximal SVC should be recognized as a potential and reversible cause of persistent abdominal contractions.

## Data Availability

All data underlying this article are included within the article. No additional datasets were generated or analysed for this case report.

## Lead author biography

Dr Sayyed Jalawan Asjad is a PGY-2 Internal Medicine Resident at SUNY Upstate Medical University, Syracuse, NY. He earned his MBBS in 2017 from Peshawar, Pakistan, and completed GMC UK registration in 2018. From March 2019 to June 2024, he worked in various roles within the UK’s NHS, obtaining the MRCP (UK) and membership of the Royal College of Physicians, Edinburgh. He moved to the USA to further his training, completing his preliminary internal medicine residency at Howard University Hospital, Washington, DC, from July 2024 to June 2025. His clinical interest is pursuing a cardiology fellowship, and he enjoys movies, hiking, and travelling.

## Notes

**Consent:** Written informed consent for publication of this case report and accompanying images was obtained from the patient or an appropriate proxy in accordance with COPE guidelines. A copy of the signed consent form is held by the treating institution and is available for review by the journal upon request.

## References

[1] Carnero-Varo A, Pérez-Paredes M, Ruiz-Ros JA, Giménez-Cervantes D, Martínez- Corbalán FR, Cubero-López T, et al “Reel syndrome”: a new form of twiddler's syndrome? Circulation 1999;100:e45–e46. 10458729 10.1161/01.cir.100.8.e45

[2] Biffi M, Exner DV, Crossley GH, Ramza B, Coutu B, Tomassoni G, et al Occurrence of phrenic nerve stimulation in cardiac resynchronization therapy patients: the role of left ventricular lead type and placement site. Europace 2013;15:77–82. 22848075 10.1093/europace/eus237

[3] Perez A, Dickey S, Fernelius J, Mar PL. An unusual case of diaphragmatic stimulation following single chamber ICD implantation. Am J Med 2023;136:e20–e21. 36170935 10.1016/j.amjmed.2022.09.011

[4] Bayliss CE, Beanlands DS, Baird RJ. The pacemaker-twiddler’s syndrome: a new complication of implantable transvenous pacemakers. Can Med Assoc J 1968;99:371–373. 4952398 PMC1924435

[5] Nicholson WJ, Tuohy KA, Tilkemeier P. Twiddler’s syndrome. N Engl J Med 2003;348:1726–1727. 12711756 10.1056/NEJM200304243481722

[6] Antonelli D, Feldman A, Freedberg NA, Turgeman Y. Transient phrenic stimulation due to twiddler syndrome. Isr Med Assoc J 2021;23:383–384. 34155855

[7] Gurkirat S, Hemant K, Zahidullah K, Narender Omprakash B. Pacemaker twiddler syndrome - A rare but serious cause of pacemaker malfunction. Int J Clin Cardiol 2018;8:ytae004.

[8] Celik T, Kose S, Bugan B, Iyisoy A, Akgun V, Cingoz F. Hiccup as a result of late lead perforation: report of two cases and review of the literature. Europace 2009;11:963–965. 19359331 10.1093/europace/eup071

